# Supplementary material for: Systems biology derived source-sink mechanism of BMP gradient formation
Source: eLife. 2017 Aug 9;6:e22199. doi: 10.7554/eLife.22199 (PMC5590806; doi:10.7554/eLife.22199)
Supplement: Supplementary file 1. [file elife-22199-supp1.zip › supplement/Image Analysis for paper/read me.docx]

IMAGE ANALYSIS INSTRUCTIONS

To start with, include the whole package (including all the subfolders) into the matlab path. You may type “pathtool” in the matlab command window, press “Add with subfolders” in the window that pops out, and then add this “Image Analysis 1.0”. You can now use all the functions, data and examples in matlab.

Step 1:

The function stacksegint3D140plus() identifies nuclei centerpoints and extracts P-Smad intensities. It outputs an array with 5 columns and X rows equal to the number of nuclei in a given embryo. Column 1 is X coordinates (in pixels), column 2 is y (in pixels), column 3 is Z slice number, column 4 is sytox green intensity, column 5 is psmad intensity. Each xy pixel is .55 um and each z slice is 2.3 um. The plotnieghbors() function then eliminates dividing cells using their sytox intensity. The array is saved as a .mat file.

Step 2:

Step 1 saves each embryo is saved as ['filename' # '_xyz']. Manually open save them together as 'NFall.mat'.

Step 3:

This step rotates the XYZ coordinates to align embryos in the Z direction and orient the ventral side to the left. It also deletes the yolk syncytial layer nuclei below the margin.

This step makes the next step’s YSL deletion easier and more accurate. This approximately translates and rotates the raw nuclei data sets as is shown in the following figure.


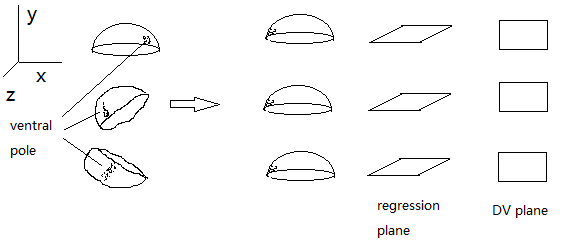
Next, the program will show a side view of each embryo and prompt the user to select 2 points. Nuclei above this line are deleted. This stet deletes the YSL nuclei below the magrin, as is shown in the following.

(before)


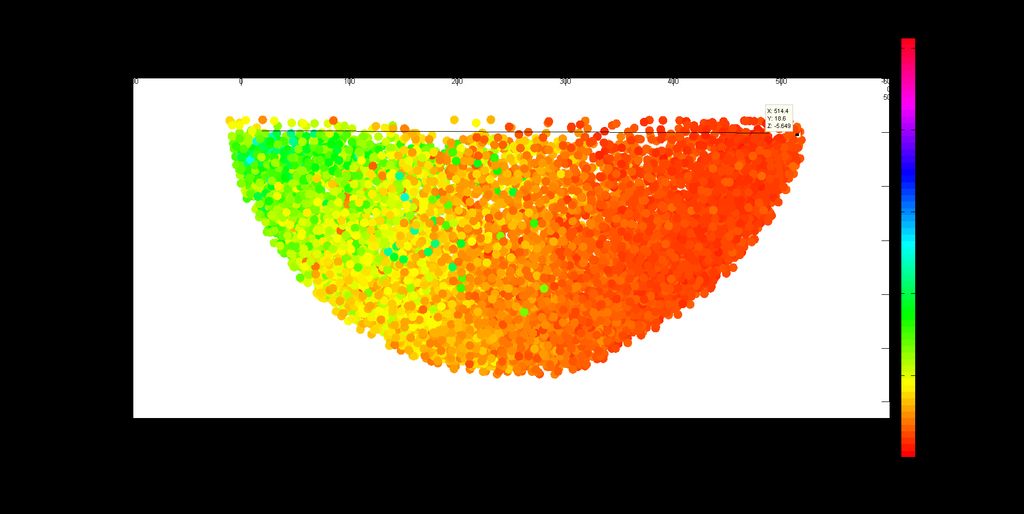


(after)


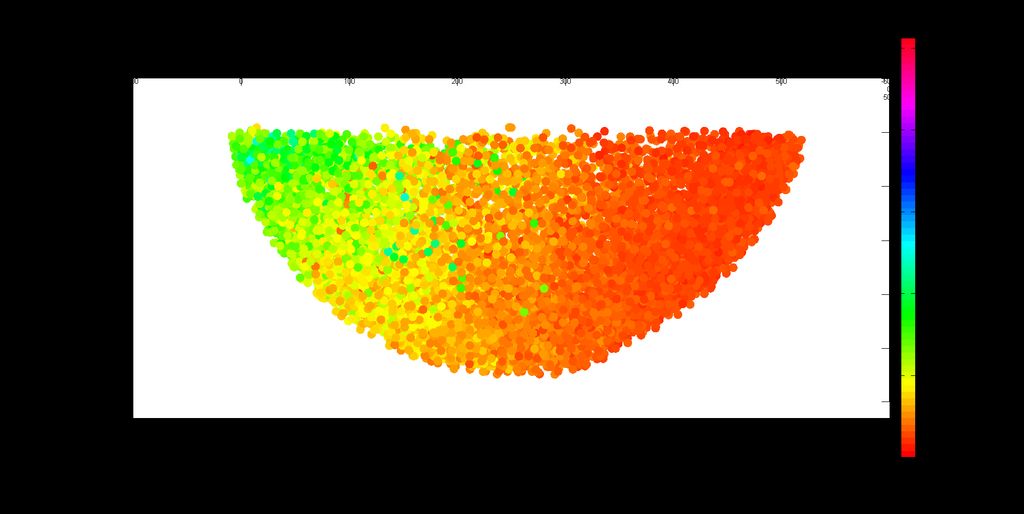


Step 4:

This registers all the nuclei data sets to a common reference embryo using affine coherent point drift. An example is in the folder “examples”. Run the example, it will first ask you to set up the C language compiler. Follow the instructions.

Step 5:

Then the outer and inner surface nuclei are deleted, as is shown in the following figure. These nuclei are potentially Enveloping layer or YSL nuclei, populations which don’t respond to P-Smad the same as deep cells from our observations.


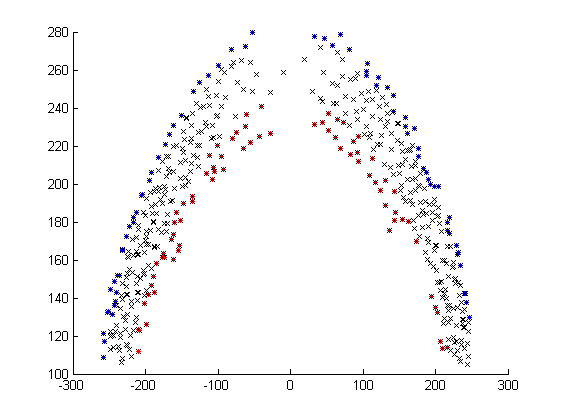


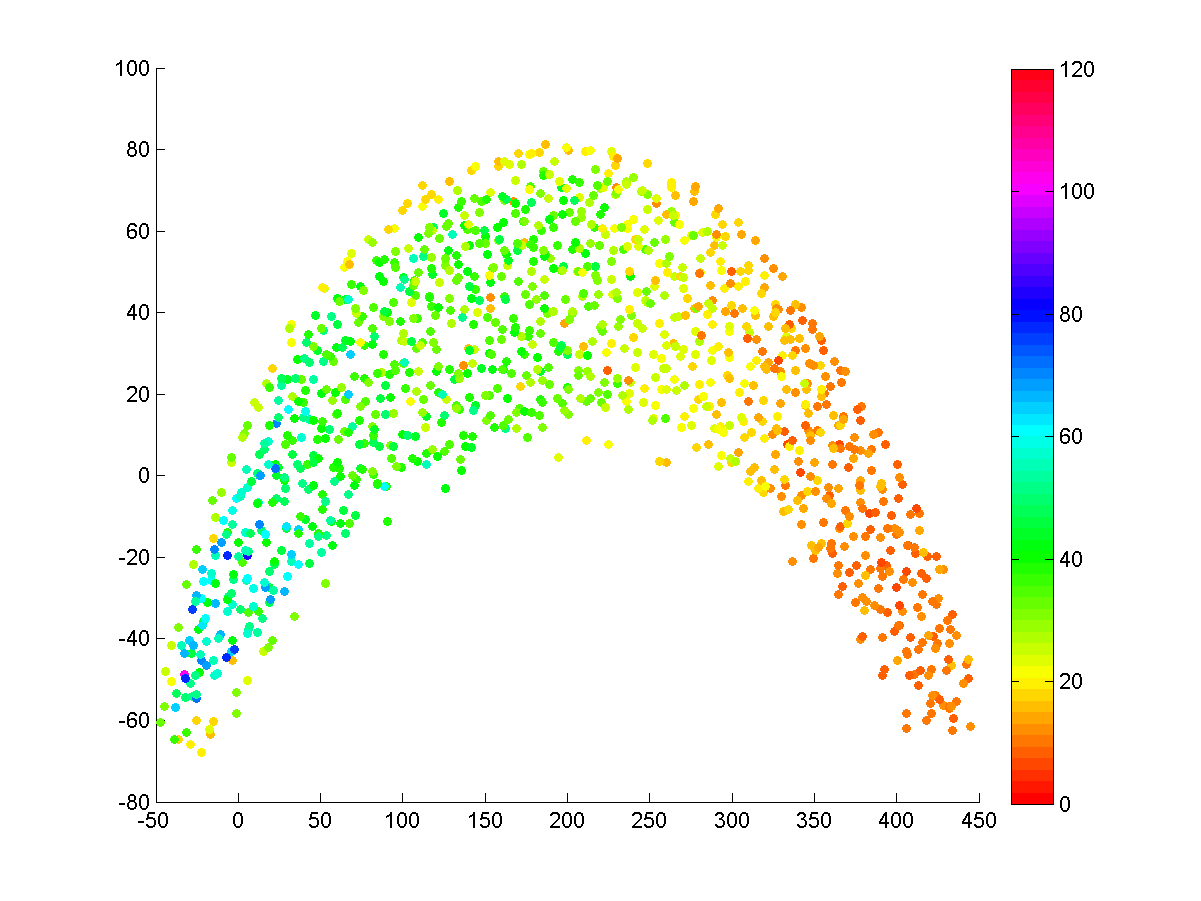

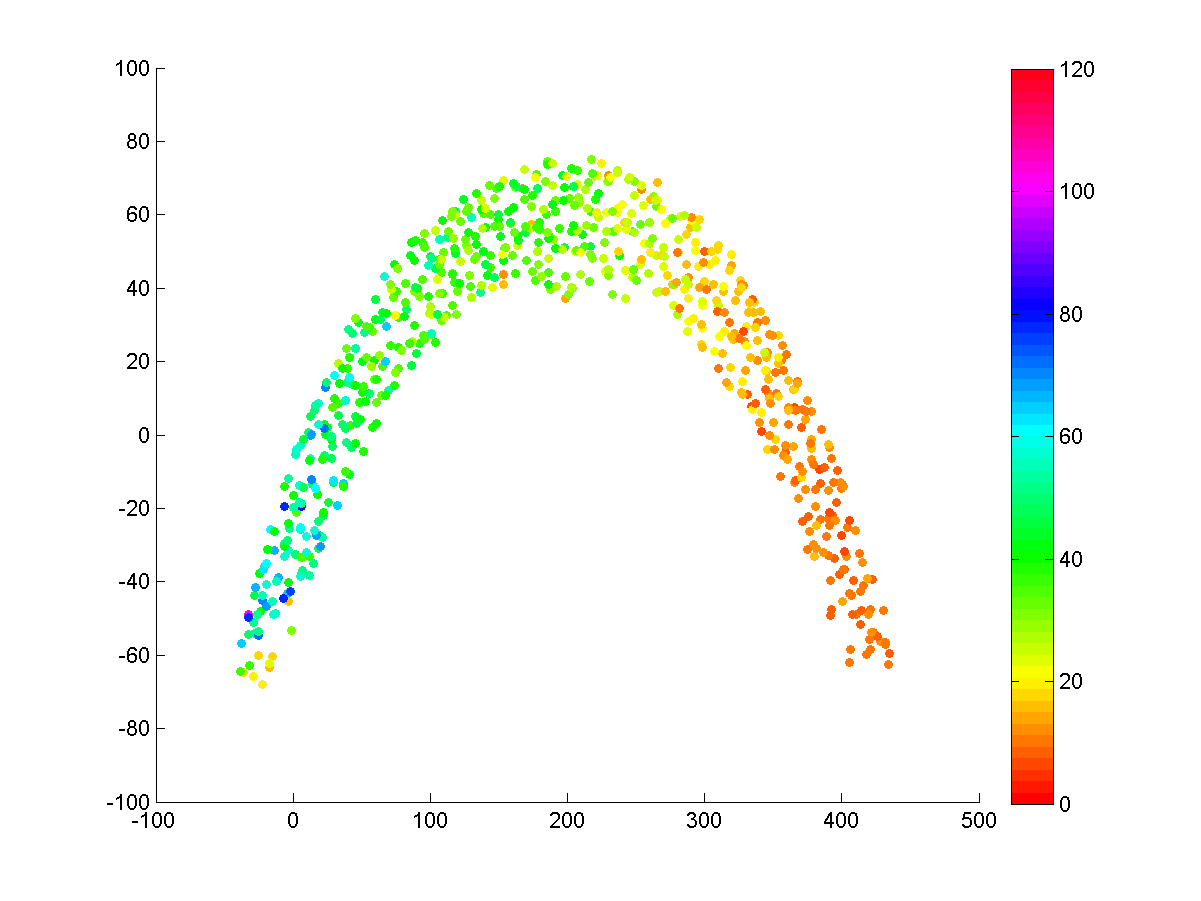


Next, all nuclei are projected onto a sphere divided into 4000+ triangles. Each triangle is an average of all nuclei within. The icosoproj() function takes in multiple alighned and registered embryos and averages all of their nuclei together for statistical analysis.

Step 6:

There are 3 built in ways to display the averaged P-Smad data. Step 6a displays all nuclei averaged together in 4000+ triangles forming a semi-spherical surface. Step 6b can compare a band of cells around the margin of two different averaged populations and perform a T-Test to test if they are significantly different. Step 6b displays the prolife of a single averaged set of embryos as well as graphs its slope.
