# Supplementary figures and images for: Systems biology derived source-sink mechanism of BMP gradient formation
Source: eLife. 2017 Aug 9;6:e22199. doi: 10.7554/eLife.22199 (PMC5590806; doi:10.7554/eLife.22199)

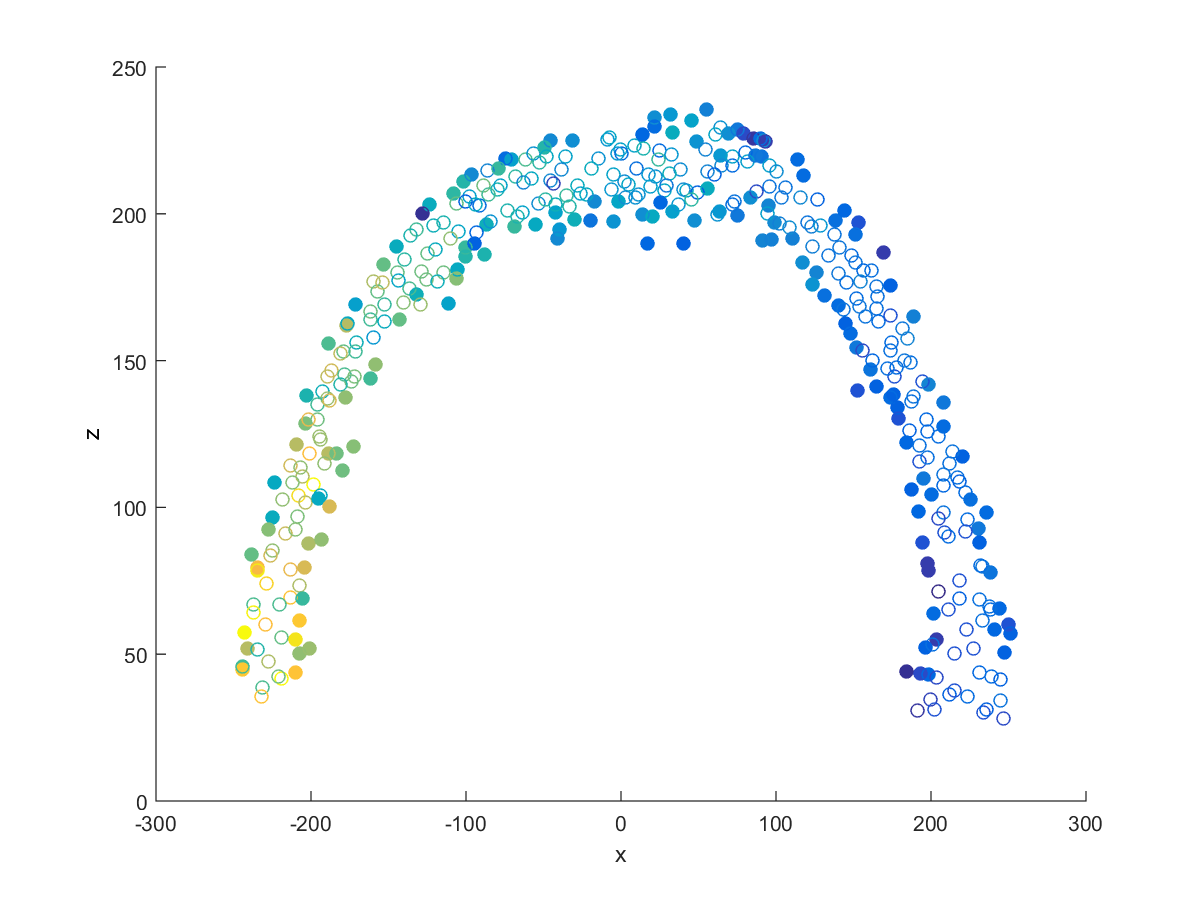

Supplement: Supplementary file 1. [file elife-22199-supp1.zip › supplement/Image Analysis for paper/nfall1_after_surface_deletion.png]

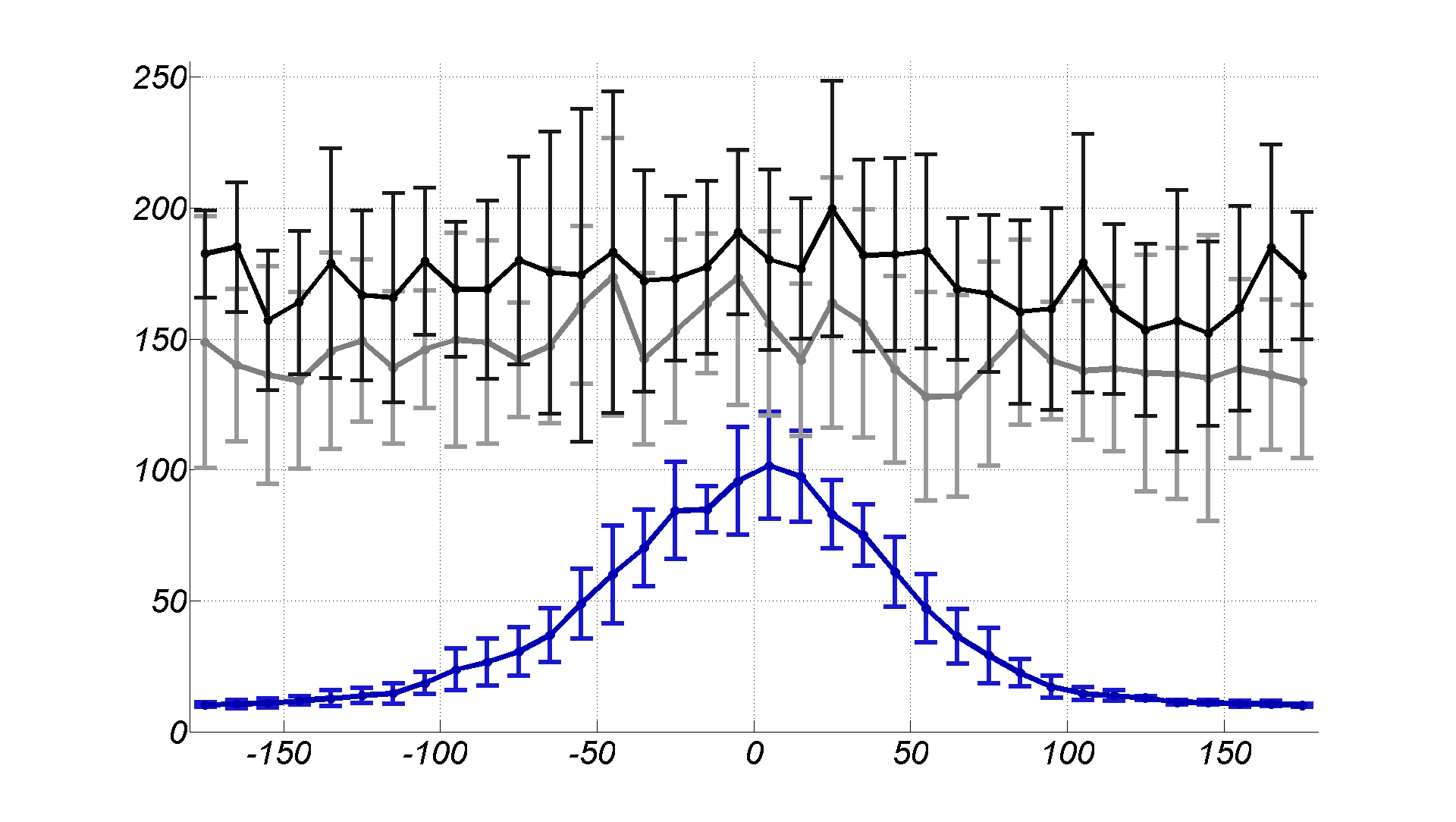

Supplement: Supplementary file 1. [file elife-22199-supp1.zip › supplement/P-Smad intensities/bmp overexpression/wtvsbmp27.tif]
